# A multi‐centric study on validation of the Fear Scale for COVID‐19 in five Arabic speaking countries

**DOI:** 10.1002/brb3.2375

**Published:** 2021-10-17

**Authors:** Hiba Deek, Mayssah El Nayal, Khalid Alabdulwahhab, Mohammad Ahmad, Riyaz Shaik, Meshari Alzahrani, Iman Elmahdi, Naglaa Youssef, Mohamed Alboraie, Daniel YT Fong, Edmond Pui Hang Choi, Bobo Kai Yin Chan, Nagla Omar

**Affiliations:** ^1^ Nursing Department, Faculty of Health Sciences Beirut Arab University Beirut Lebanon; ^2^ Faculty of Health Sciences, Beirut Arab University Beirut Lebanon; ^3^ Department of Ophthalmology College of Medicine, Majmaah University Al Majmaah 11952 Saudi Arabia; ^4^ Department of Community Medicine and Public Health College of Medicine, Majmaah University Al Majmaah 11952 Saudi Arabia; ^5^ Department of Urology College of Medicine, Majmaah University Al Majmaah 11952 Saudi Arabia; ^6^ Faculty of Pharmacy, University of Benghazi; ^7^ Medical‐Surgical Nursing Department, Faculty of Nursing Cairo University Egypt; ^8^ Department of Internal Medicine Al‐Azhar University Cairo Egypt; ^9^ School of Nursing The University of Hong Kong; ^10^ Community Medicine Department Alzaiem Alazhari University Faculty of Medicine

**Keywords:** Arabic, fear, psychometrics, questionnaire, reliability, validity

## Abstract

**Background:**

The Eight‐item Fear Scale is a unidimensional scale evaluating the perceived feelings of fear associated with the thought of the coronavirus.

**Aim:**

The Arabic version of this scale did not exist; hence, this study aimed to translate and evaluate the psychometric properties of the Fear Scale in participants aged 18 years and above in five Arabic countries: Egypt, Lebanon, Libya, Saudi Arabia, and Sudan by using a cross‐sectional survey design.

**Method:**

The English version of the COVID‐19 Fear Scale was translated into Arabic following the guidelines and disseminated through social media. Factorial and convergent validity and internal reliability were evaluated. Results: The total number of participants was 2783; the majority was young (41.9%) and female (60.5%). Fear scores were moderate in four countries and severe in Egypt. The scale showed good structural validity, with the items explaining up to 70% of the variance. The scale items correlated significantly with the total scores, and the Cronbach alpha was above 0.9.

**Conclusion:**

The study concluded that the Arabic Fear Scale is a psychometrically robust scale that can be used to evaluate the perceived feelings of fear with the thought of the coronavirus or pandemic in general.

## Abstract 2

(1) This study aimed to translate and evaluate the psychometric properties of the Fear Scale in above 18 years Arabic speakers’ residents in five Arabic countries: Egypt, Lebanon, Libya, Saudi Arabia and Sudan. (2) It used a cross‐sectional survey design, the English version of COVID‐19 Fear Scale was translated in Arabic following the guidelines and disseminated through social media. Structural and Criterion validity, internal consistency, and reliability were evaluated. (3) The study concluded that evaluating fear in the Arabic countries was found to be essential considering the high scores.

## 1 INTRODUCTION

On December 31, 2019, the WHO Regional Office in China was informed of cases of pneumonia of unknown cause detected in Wuhan City, Hubei Province of China (World Health Organization, 2020a). On January 7, 2020, the Chinese authorities reported having identified a novel virus that caused these cases (World Health Organization, 2020b). The COVID‐19 outbreak was declared a global pandemic on March 11, 2020, and is an unprecedented public health emergency of this century (Centers for Disease Control and Prevention, 2020). At this point, COVID‐19 had developed into a pandemic associated with substantial morbidity and mortality. By the end of 2020, 61.8 million reported cases and 1.4 million deaths had been identified worldwide (World Health Organization, 2020d). The MENA region accounted for 4.6% of the global burden of COVID‐19 cases and 2.9% of the global COVID19‐associated mortalities (IOM UN Migration, 2020).

To slow the transmission and to avoid associated disease, pandemic mitigation steps were laid down. These measures included complete or partial lockout, travel prohibitions, limitations on mass collection, neighborhood home quarantines, physical distance measures, personal security measures, and other non‐pharmaceutical interventions. In addition, this unpredictable and increasingly spreading infectious disease has triggered widespread awareness, anxiety, and depression, all of which are normal psychological reactions to the randomly evolving situation, according to the WHO (World Health Organization, 2020c).

The prevailing situation of pandemics leading to substantial morbidity and mortality, coupled with economic impact because of the lockdown enforced in many countries, has resulted in adverse psychological outcomes in the community. Based on the literature, fear is impregnated in different sections of society in response to contracting the disease, and job losses across various sectors are causing marked impairment in daily life functioning. Daily activities or situations like meeting people, traveling, and excessive information in social media serve as the triggering factors for people to develop fear. Psychological effect in the form of fear may affect all age groups including children, as reported by some studies during the H1N1 swine‐flu pandemic of 2009. Children's fear of reactions about the disease was found to be correlated with H1N1‐related threat information from parents or social media (Remmerswaal & Muris, 2011). Adults with co‐morbidities are susceptible to severe outcomes, with some requiring intensive care. This, in turn, has also contributed considerably to the development of irritability, anxiety, and fear (Muris & Field, 2010). Health care professionals in India, working long hours with COVID‐19 patients, were reported to have health crimes originating from the fear of contracting the virus (The Times of India, 2020).

Various scales apart from the commonly used scales like GAD‐7 (Al‐Rabiaah et al., 2020) to measure anxiety were also developed to evaluate the psychological impact and fear. COVID‐19 stress scale was developed by Taylor et al. (2020), for the measurement of stress and anxiety, with emphasis on fear due to contamination, economic consequences, xenophobia, and traumatic stress symptoms. Another scale was developed by Schimmenti et al. (2020) with the conceptual analysis of four domains of fear, namely, fear of the body, fear of not knowing, fear of significant others, and fear of inaction. Some of the studies carried out during the early phase of the COVID‐19 pandemic assessed the psychological impact using the impact of Event Scale‐Revised (Zhang et al., 2014), and mental health status was assessed by the Depression, Anxiety and Stress Scale (DASS‐21). The Fear of COVID‐19 is another scale developed by Ahorsu et al. (2020) to evaluate fear associated with contracting the virus. This same seven‐item scale was validated in many languages and lastly in the Amharic language to study its psychometric properties in the Ethiopian population (Elemo et al., 2020). Fear is a subjective response and can vary in different ethnic people; an attempt has been made to measure the fear in five Arab counties, with a scale validated and tested in the Arabic language.

This study was part of an extensive international survey aimed to evaluate the effect of the COVID‐19 pandemic on health, fear, and depression. The more comprehensive study was explained elsewhere (Lok et al., 2021) and briefed here.

### 1.1 Current study's aim

This study aimed to translate the English version of the Fear Scale into Arabic and to evaluate its psychometric properties in a large sample of Arabic‐speaking individuals from five Arabic countries (Egypt, Lebanon, Libya, Saudi Arabia, and Sudan).

## 2 METHODS

### 2.1 Linguistic evaluation of the Fear Scale

To translate and validate the Arabic version of the Fear Scale, steps recommended by Anastasi's (Anastasi, 1985) and Abdel‐Khalek (Abdel‐Khalek & Snyder, 2007) were adopted as follows.

#### 2.1.1 Forward translation

Two bilinguals and fluent English and Arabic speakers independently translated the original Fear Scale from English to Arabic. Then, a consensus meeting comprising the two translators and other Arabic‐speaking team members reviewed the two Arabic‐translated versions of the scales and discussed discrepancies. With this, a consensus Arabic version was obtained.

#### 2.1.2 Back‐translation

The consensus Arabic version of the scale was then back‐translated to English by a third translator who was unaware of the original English version. Finally, the two English versions were reviewed by a native English speaker who had prior experience of the cultural adaptation process. No discrepancies were observed.

#### 2.1.3 Panel expert revision and content validity

For a linguistic adaptation of the Arabic Fear Scale, a panel expert of five Arabic‐speaking researchers specialized in psychology, nursing, and public health were involved. Those who are bilingual and fluent in Arabic and English were asked to review the content of the Arabic version to obtain their opinions regarding the appropriateness, adequacy, and content validity of the Arabic version. The panel validated the wording and sentence structure and approved the final version.

#### 2.1.4 The pilot for the cognitive debriefing of the Arabic Fear Scale

The penultimate version of the Arabic Fear Scale was piloted with six individuals from the five participated countries. They were invited to complete the Arabic Fear Scale sent electronically, i.e., email, WhatsApp, etc., alongside an evaluation form. The evaluation form was designed to elicit the Arabic Fear Scale clarity, relevancy, and understandability. It included these items: completion time, estimating the length of the scale, relevance to condition, list of irrelevant items, overall clarity, list of unclear items, and open‐ended questions for other vital issues that can be added or modified in the scale allowing the participants to elaborate on their comments on the items of the scale in lay terms. The pilot analysis showed that the median time for completion was 1 min (range 1 to 3 min). All participants considered the scale to have an acceptable or short length with moderate to high relevance. Five (83.3%) thought the scale to have high or very high clarity. The expert panel that reviewed the responses found that no substantial issues were raised. The final version is attached in Appendix A (Supporting Information).

### 2.2 Psychometric evaluation of the Fear Scale

This study was part of the international CARE project, which aimed to identify the causes of fear, fear levels, anxiety, and depression in participants recruited from over 30 countries (Lok et al., 2021). Data collection was done through a cross‐sectional self‐administered questionnaire between August and December 2020.

#### 2.2.1 Eligibility criteria

Eligible participants were community members aged 18 years or above, free from any psychological or physical problems affecting communication, and residents of one of the study countries: Egypt, Lebanon, Libya, Saudi Arabic, and Sudan.

#### 2.2.2 Sample size calculation

In the CARE project, the sample size calculation was based on estimating the prevalence of a health issue. Taking the most conservative scenario of 50%, with a 5% margin of error in a 95% confidence interval, 385 subjects were needed from each country. To account for incomplete responses, 500 subjects were targeted per country. In our application, the evaluation of the factorial validity of the 8‐item Fear Scale would require 100 subjects by the rule of thumb of 80 subjects per item (Kline, 2015). Hence, the sample size should be adequate for the psychometric evaluation.

### 2.3 Instruments

Data were collected using an anonymous electronic survey that included four sections: (1) Sociodemographic variables [i.e., age, gender, marital status, education, occupation], (2) medical history data [i.e., diagnosed by the doctor with any medical conditions] (World Health Organization, 2014), (3) COVID‐19 contraction in their immediate community, and (4) Patient Health Questionnaire (PHQ‐4), and (5) the Arabic version of the Fear Scale.

#### 2.3.1 Patient Health Questionnaire

PHQ‐4: This is a four‐item questionnaire with two questions that evaluate anxiety and another two evaluate depression. This questionnaire showed strong psychometric properties with 84% explained variance on factor analysis and a Cronbach alpha of 0.8 (Kroenke et al., 2009). The PHQ‐4 was previously translated to Arabic and validated in an Arabic speaking sample (Kliem et al., 2016).

#### 2.3.2 Fear Scale

The Fear Scale was adapted from the Champion Breast Cancer Fear Scale (CBCFS) (Champion et al., 2004). It is an eight‐item unidimensional scale for evaluating the perceived feeling of fear associated with the thought of the coronavirus. The original scale showed that the eight items fell under the concept of fear that explained 57% of the variance in exploratory factor analysis (EFA). The Cronbach alpha of the CBCFS was 0.91 (Champion et al., 2004). Each item responded on a 5‐point Likert scale, and a higher total score indicated a higher fear level. There had been no cut‐off point for defining high or low fear. Prior approval for translating the Fear Scale from English to Arabic has been sought from the developer.

### 2.4 Setting

The study was carried out in five countries in the Middle East, including Egypt, Lebanon, Libya, Saudi Arabia, and Sudan.

### 2.5 Ethical considerations and data protection

After securing ethical approval from the Institutional Review Boards of the five respective countries, an electronic, anonymized self‐administered questionnaire was generated using a secured survey platform. The first cover page of the questionnaire explained that the survey was related to the COVID‐19 outbreak. The Informed Consent was displayed on the front page of the survey that required the respondent to agree to a consent statement before proceeding with the survey. The respondents were assured that participation in the study was voluntary and that they could withdraw from the study anytime without any penalty, and all the information would be kept confidential, and results would be reported in aggregate form.

### 2.6 Recruitment process

The link generated by the electronic platform was circulated among the acquaintances of the research team in each country. Social media and mobile applications such as WhatsApp were used for disseminating the link.

### 2.7 Data analysis

Descriptive statistics were performed on the eight items of the scale. To examine the factorial structure of the scale, EFA was conducted with factor loadings estimated by principal axis factoring. We adopted EFA since the structure of the Fear Scale for COVID‐19 has not been empirically examined. The elbow method on the scree plot was used to determine the number of factors, and the rotation method used was promax. The KMO determined the sampling adequacy, and Bartlett's test of sphericity was used to indicate the significant corrections within the variables and the matrix. Floor and ceiling percentages were calculated to measure the sensitivity and coverage of the Fear Scale total score at each end (Gulledge et al., 2019). Convergent validity was assessed by examining the association between the Fear Scale and the PHQ‐4 by using the Spearman rank correlation. This was conducted with the assumption that fear would be associated with depression and anxiety (Hosseini & Khazali, 2013). To assess the internal reliability, the item‐total correlation and the Cronbach alpha were calculated. A *p*‐value less than .05 was considered statistically significant. Statistical Product and Service Solutions (SPSS) v. 26 were used to perform this analysis.

## 3 RESULTS

### 3.1 Sociodemographic characteristics of the participants

A total of 2783 participants (497 from Egypt; 459 from Lebanon; 654 from Libya; 627 from Saudi Arabia, and 546 from Sudan) were included in this study. The majority of the participants were aged between 18 and 24 years. This age group was the highest in all the study countries, with the highest reaching 70% in Sudan. Almost two‐fifths of the sample were male participants, and the majority (*n* = 1650, 59.3%) were single. More than half were Bachelor degree holders across the five countries, with 38.5% being students. More than 70% had never consumed alcohol, and a similar number reported never smoking a cigarette. In terms of physical activity, almost half the sample reported never being involved in vigorous exercises. This was reflected in the mean hours of 8.51 (SD = 4.65) of sitting time and 7.84 (SD = 4.52) of screen time per day. The mean number of people living in the same household was 5.81 (SD = 3.24), with the highest being in Sudan and the lowest in Egypt. In terms of medical conditions, the highest prevalence was for irritable bowel disease (7.4%), and the lowest being for stroke with only one case in Saudi Arabia, while only 27.5% reported the following up on their medical condition. Table 1 summarizes the sample characteristics. Most of the study participants reported having a good knowledge level about the coronavirus (87.4%)and a good knowledge level on the prevention of its spread (90.5%), while more than half (57.6%) reported being susceptible to contracting the virus. Two thirds (66%) reported that the virus spread was rather severe in their community, although only 352 (12.6%) reported contracting the virus. However, 67.9% reported that someone in their immediate environment contracted the virus. The details of the participants' knowledge of the COVID‐19 are presented in Table 2.

**TABLE 1:** Sociodemographic characteristics of the study participants (*N* = 2783)

Variables	Total sample (*n* = 2783, 100%)	Egypt (*n* = 497, 17.9%)	Lebanon (*n* = 495, 16.5%)	Libya (*n* = 654, 23.5%)	Saudi Arabia (*n* = 627, 22.5%)	Republic of Sudan (*n* = 546, 19.6%)	*p*‐value
Age							
18–24	1165 (41.9)	105 (21.1)	215 (46.8)	245 (37.5)	218 (34.8)	382 (70)	<.001
25–29	468 (16.8)	75 (15.1)	46 (10)	143 (21.9)	118 (18.8)	86 (15.8)	
30–34	350 (12.6)	98 (19.7)	55 (12)	92 (14.1)	85 (13.6)	20 (3.7)	
35–39	298 (10.7)	95 (19.1)	52 (11.3)	72 (11)	70 (11.2)	9 (1.6)	
40–44	188 (6.8)	58 (11.7)	30 (6.5)	41 (6.3)	46 (7.3)	13 (2.4)	
45–49	130 (4.7)	18 (5.6)	20 (4.4)	22 (3.4)	47 (7.5)	13 (2.4)	
50–54	102 (3.7)	23 (4.6)	18 (3.9)	23 (3.5)	24 (3.8)	14 (2.6)	
55–59	47 (1.7)	8 (1.6)	14 (3.1)	11 (1.7)	10 (1.6)	4 (0.7)	
60–64	26 (0.9)	4 (0.8)	7 (1.5)	4 (0.6)	7 (1.1)	4 (0.7)	
≥65	9 (0.3)	3 (0.6)	2 (0.4)	1 (0.2)	2 (0.3)	1 (0.2)	
Female	1685 (60.5)	284 (57.1)	301 (65.6)	379 (58)	373 (59.5)	348 (63.7)	.019
Social status							
Single	1650 (59.3)	177 (35.6)	265 (57.7)	423 (64.7)	322 (51.4)	463 (84.8)	<.001
Married	1056 (37.9)	302 (60.8)	179 (39)	216 (33)	284 (45.3)	75 (13.7)	
Divorced/widow/separated	77 (2.8)	18 (3.6)	15 (3.3)	15 (2.3)	21 (3.3)	8 (1.5)	
Level of education							
≤Elementary	20 (0.7)	2 (0.4)	7 (1.5)	3 (0.5)	5 (0.8)	3 (0.5)	<.001
≥High school education	2763 (99.3)	2781 (99.6)	2776 (98.5)	2780 (99.5)	2778 (99.2)	2780 (99.5)	
Occupation							
Unemployed	361 (9.4)	26 (5.2)	64 (13.9)	49 (7.5)	80 (12.8)	42 (7.7)	<.001
Employed	2422 (90.6)	2757 (94.8)	2719 (86.1)	2734 (92.5)	2703 (87.2)	2741 (92.3)	
Alcohol consumer	101 (3.6)	478 (96.2)	51 (11.1)	20 (3.4)	2 (0.3)	9 (1.7)	
Smoker	523 (18.8)	61 (12.3)	188 (40.9)	114 (17.7)	104 (16.6)	56 (10.2)	<.001
Physical activity	1928 (69.3)	344 (69.2)	323 (70.4)	442 (67.9)	460 (73.4)	359 (65.8)	.490
Sitting time (h)	8.51 (4.65)	8.28 (4.02)	8.38 (4.12)	8.17 (4.73)	8.74 (4.85)	8.98 (5.19)	.017
Screen time (h)	7.84 (4.52)	7.10 (3.91)	7.25 (3.92)	7.93 (4.59)	8.20 (4.63)	8.58 (5.16)	<.001
Health care professional	957 (34.4)	221 (44.5)	181 (39.4)	220 (33.6)	155 (24.7)	180 (33)	<.001
Perceived social rank^a^							
1 (do not have enough money/go to the worst schools, might not have a job, don't live in a nice place)	180 (6.5)	18 (3.6)	26 (5.7)	60 (9.2)	25 (4)	51 (9.3)	<.001
2	266 (9.6)	44 (8.9)	58 (12.6)	68 (10.4)	49 (7.8)	47 (8.6)	
3	1341 (48.2)	257 (51.7)	271 (59)	289 (44.2)	256 (40.8)	268 (49.1)	
4	680 (24.4)	120 (24.1)	91 (19.8)	143 (21.9)	202 (32.2)	124 (22.7)	
5 (have the best jobs/go to the best schools, have lots of money, live in nice places)	316 (11.4)	58 (11.7)	13 (2.8)	94 (14.4)	95 (15.2)	56 (10.3)	
Children below 18	883 (31.7)	266 (53.5)	140 (30.5)	175 (26.8)	248 (39.6)	54 (9.9)	<.001
Number of children below 18	2.43 (1.74)	2.20 (2.35)	1.95 (1.16)	2.83 (1.32)	2.69 (1.39)	2.73 (1.37)	<.001
Number of people in the household	5.81 (3.24)	4.64 (1.84)	4.58 (1.53)	6.24 (3.13)	6.41 (3.64)	6.80 (4.24)	F(4) = 1.407
Medical diagnosis							
Anxiety	168 (6)	40 (8)	35 (7.6)	32 (4.9)	44 (7)	17 (3.1)	<.001
Depression	121 (4.3)	34 (6.8)	22 (4.8)	16 (2.4)	28 (4.5)	21 (3.8)	<.001
Cardiovascular diseases	278 (10.1)	84 (16.9)	62 (13.6)	40 (6.1)	70 (11.2)	22 (4)	
Respiratory problems (COPD, asthma, etc.)	54 (1.9)	4 (0.8)	13 (2.8)	10 (1.5)	12 (1.9)	15 (2.7)	.100
Blood sugar (pre‐diabetes and diabetes)	137 (4.9)	31 (6.2)	19 (4.1)	25 (3.8)	41 (6.5)	20 (3.6)	
Nephropathy	21 (0.8)	3 (0.6)	1 (0.2)	6 (0.9)	6 (1)	5 (0.9)	.610
Cancer	10 (0.4)	4 (0.8)	3 (0.7)	0 (0)	3 (0.5)	0 (0)	.070
Other	640 (23)	181 (36.4)	113 (24.7)	148 (22.8)	122 (19.4)	76 (13.9)	
Proper medical follow up	764 (27.5)	128 (25.8)	165 (35.9)	198 (30.3)	184 (29.3)	89 (16.3)	<.001

^a^ Data presented in means and standard deviation.

^b^Non‐university degree.

^c^Significant at .001.

**TABLE 2:** Participants’ knowledge toward the COVID‐19 (*n* = 2783)

	Total sample (*n* = 2783, 100%)	Egypt (*n* = 497, 17.9%)	Lebanon (*n* = 495, 16.5%)	Libya (*n* = 654, 23.5%)	Saudi Arabia (*n* = 627, 22.5%)	Republic of Sudan (*n* = 546, 19.6)	*p*‐Value
How would you rate your knowledge level on the novel coronavirus?							
Poor	96 (3.5)	11 (2.2)	11 (2.4)	21 (3.3)	24 (3.8)	29 (5.2)	<.001
Neutral	251 (9)	48 (9.7)	39 (8.5)	57 (8.7)	51 (8.1)	56 (10.3)	
Good	2434 (87.4)	438 (88.2)	409 (89.2)	574 (87.8)	552 (88)	461 (84.5)	
How would you rate your knowledge level on how to prevent the spread of the novel coronavirus?							
Poor	78 (2.8)	12 (2.4)	25 (5.5)	19 (3)	17 (2.7)	15 (4.5)	<.001
Neutral	185 (6.7)	49 (9.9)	50 (10.9)	42 (6.4)	30 (4.8)	44 (8.1)	
Good	2468 (90.5)	436 (87.8)	384 (83.7)	591 (90.4)	580 (92.5)	477 (87.3)	
Do you think you have adequate knowledge about the novel coronavirus?							
Inadequate	260 (9.4)	43 (8.6)	22 (4.8)	69 (10.5)	43 (6.9)	83 (15.2)	<.001
Neutral	229 (8.2)	40 (8)	41 (8.9)	45 (6.9)	53 (8.5)	50 (9.2)	
Adequate	2292 (82.3)	414 (83.3)	396 (86.3)	538 (82.2)	531 (84.7)	413 (75.7)	
During the pandemic, how susceptible do you consider yourself to an infection with the novel coronavirus?							
Not susceptible	721 (25.9)	95 (19)	169 (36.8)	175 (26.7)	170 (20.4)	112 (20.5)	< .001
Neutral	457 (16.4)	80 (16.1)	58 (12.6)	106 (16.2)	135 (5.6)	78 (14.3)	
Susceptible	1603 (57.6)	322 (64.8)	232 (50.5)	371 (56.7)	322 (51.4)	356 (65.2)	
During the pandemic, how severe would contracting the novel coronavirus be for you?							
Not severe	1177 (42.3)	117 (23.6)	214 (46.6)	310 (47.4)	289 (46.1)	247 (45.3)	<.001
Neutral	654 (23.5)	145 (29.2)	93 (20.3)	141 (21.6)	170 (27.1)	105 (19.2)	
Severe	950 (34.1)	235 (47.2)	12 (33.1)	201 (30.7)	168 (26.9)	194 (35.5)	
During the pandemic, how severe is the spread of the novel coronavirus in your community?							
Not severe	655 (10.3)	51 (10.2)	101 (22)	147 (22.4)	130 (23.2)	154 (28.2)	< .001
Neutral	290 (10.4)	57 (11.5)	43 (9.4)	68 (10.4)	81 (12.9)	41 (7.5)	
Severe	1836 (66)	389 (78.3)	315 (68.4)	437 (66.8)	344 (54.8)	351 (64.3)	
Are you or have you been infected with the coronavirus?							
Yes	352 (12.6)	91 (18.3)	22 (4.8)	95 (14.6)	63 (10)	81 (14.8)	<.001
No	2022 (72.7)	294 (59.2)	296 (86.3)	456 (69.7)	511 (81.5)	365 (66.8)	
Don't know	407 (14.6)	112 (22.5)	41 (8.9)	101 (99.7)	53 (8.5)	100 (18.3)	
Do you know people in your immediate social environment who are or have been infected with the novel coronavirus?							
Yes	1889 (67.9)	379 (76.3)	250 (54.5)	436 (66.8)	480 (76.6)	344 (63)	<.001
No	762 (27.4)	98 (19.7)	189 (41.2)	177 (27.1)	131 (20.9)	167 (30.6)	
Don't know	130 (4.7)	20 (4)	20 (4.4)	39 (6)	16 (2.6)	35 (6.4)	
Fear Scale*	22.19 (7.872)	23.73 (7.39)	22.39 (7.79)	22.07 (7.95)	21.30 (8.21)	21.80 (7.67)	<.001

*: data presented in means and standard deviation.

### 3.2 Fear among the participants

The fear scores varied significantly across the countries (F(4) = 7.23; *p* < .001) where the highest scores were in Egypt (mean score = 23.73), followed by Lebanon (22.39), Libya (22.07), Sudan (21.80), and Saudi Arabia (21.30). The minimum and maximum scores were 8 and 40, respectively, with a mean score of 22.19 across the five countries (SD = 7.87). Moreover, the floor and ceiling effects were 6.4% and 2%, respectively, with only 0.01 missing values.

### 3.3 Exploratory factor analysis

Table 3 shows the EFA of the Fear Scale. The KMO value (>0.919) and the significance of Bartlett's test indicated that EFA was adequate in all five countries. The one‐factor model was consistently obtained in the overall sample as well as in all the five countries, with the total variance explained in a range between 65.029 in Libya and 71.045 in Egypt. There was no substantial difference in the factor loadings across the five countries.

**TABLE 3:** Factor structure, internal construct validity, and reliability of the Fear Scale (*n* = 2783)

Fear Scale items	Factor loading of EFA	Item‐total correlation	Factor loading of EFA	Item‐total correlation	Factor loading of EFA	Item‐total correlation	Factor loading of EFA	Item‐total correlation	Factor loading of EFA	Item‐total correlation	Factor loading of EFA	Item‐total correlation
	Total sample		Egypt		Lebanon		Libya		Saudi Arabia		Sudan	
1. The thought of COVID‐19 scares me	0.662	.707^a^	0.681	0.715^a^	0.704	0.748^a^	.632	0.677^a^	0.705	0.750^a^	0.596	0.655^a^
2. When I think about COVID‐19, I feel nervous	0.807	0.814^a^	0.850	0.852^a^	0.840	0.827^a^	0.775	0.781^a^	0.821	0.831^a^	0.768	0.785^a^
3. When I think about COVID‐19, I get upset	0.840	0.842^a^	0.884	0.875^a^	0.872	0.862^a^	0.836	0.842^a^	0.821	0.833^a^	0.806	0.814^a^
4. When I think about COVID‐19, I get depressed	0.849	0.858^a^	0.865	0.862^a^	0.856	0.857^a^	0.835	0.847^a^	0.874	0.884^a^	0.811	0.834^a^
5. When I think about COVID‐19, I get jittery	0.900	0.894^a^	0.908	0.904^a^	0.924	0.909^a^	0.896	0.893^a^	0.907	0.901^a^	0.871	0.864^a^
6. When I think about COVID‐19, my heart beats faster	0.784	0.803^a^	0.819	0.837^a^	0.779	0.775^a^	0.790	0.805^a^	0.757	0.786^a^	0.780	0.800^a^
7. When I think about COVID‐19, I feel uneasy	0.832	0.841^a^	0.840	0.842^a^	0.852	0.864^a^	0.821	0.833^a^	0.842	0.854^a^	0.816	0.825^a^
8. When I think about COVID‐19, I feel anxious	0.862	0.866^a^	0.876	0.874^a^	0.888	0.892^a^	0.840	0.847^a^	0.877	0.881^a^	0.840	0.850^a^
Variance explained (EFA)	67.239		71.045		70.863		65.029		68.527		62.393	
KMO	0.934		0.936		0.939		0.925		0.925		0.919	
Bartlett's test	*X* ^2^ = 18579.600, df = 28, *p* < .001		*X* ^2^ = 3724.545, df = 28, *p* < .001		*X* ^2^ = 3435.059, df = 28, *p* < .001		*X* ^2^ = 4104.573, df = 28, *p* < .001		*X* ^2^ = 4416.588, df = 28, *p* < .001		*X* ^2^ = 3161.816, df = 28, *p* < .001	
Cronbach's alpha	0.942		0.951		0.950		0.936		0.945		0.928	

Abbreviations: COVID‐19, Coronavirus disease; df, degrees of freedom; EFA, Exploratory factor analysis; KMO, Kaiser–Meyer–Olkin.

^a^ Significant at less than .001.

#### 3.3.1 Convergent validity

Correlation coefficient analysis proved that the Fear Scale moderately correlated with the PHQ‐4 scale (Spearman rank correlation = 0.368, *p* < .001).

#### 3.3.2 Internal reliability analysis

All item‐total correlation was significant and was at least 0.66 in the overall sample as all the five countries (*p* < .001). The Cronbach alpha of the Fear Scale score for Egypt was 0.951, Lebanon was 0.950, Libya was 0.936, Saudi Arabia was 0.945, and Sudan was 0.928.

## 4 DISCUSSION

This was the first study that evaluated the psychometric properties of the Arabic version of the Fear Scale for COVID‐19. This was done across five Arabic‐speaking countries on a sample of 2783 participants. The Fear Scale showed good validity and reliability scores in each of the five samples, as was shown in the previous evaluations of the scale on a sample of 1390 women evaluated for having a fear of acquiring breast cancer (Champion et al., 2004). Furthermore, the level of fear was comparable to the current study population showing the perceived threat of the community from the COVID‐19 and the consequences of contracting the virus.

The Fear Scale is exhibited as a unidimensional scale for assessing the fear level of COVID‐19. It was demonstrated by a clear one‐factor structure irrespective of the whole sample or in each of the five Arabic speaking countries. The variance explained by a single factor was at least 62% which is higher than the reported 48% in the original English scale for assessing the fear level in breast cancer patients (Champion et al., 2004). Moreover, all items were strongly associated with the total score in the range of 0.66 to 0.90 which was again generally higher than the range of 0.46 to 0.78 in the original English version, despite the latter was corrected for overlap. In addition, the internal consistency is high with the range of 0.93 to 0.95, which is slightly higher than the 0.91 for the original English version. Hence, a single construct underlying the Fear Scale is affirmative.

The Fear Scale also demonstrated satisfactory convergent validity by a moderate association with the PHQ‐4 that collectively assesses anxiety and depression. In our context, fear is specific to the threat of COVID‐19, and both anxiety and depression are generalized reactions to this threat (Champion et al., 2004). Therefore, fear was hypothesized to be associated with anxiety and depression, despite they assess distinct constructs. On the other hand, the low floor and ceiling percentages show that Fear Scale possesses reasonable sensitivity to discriminate the fear level over its plausible range.

According to the Fear Scale, fear scores ranging between 16 and 23 are considered moderate fear scores (Champion et al., 2004). Therefore, all but one Arabic country exhibited reasonable fear of the COVID‐19 while Egypt reached the severe score, followed by Lebanon, which edged extreme fear. Furthermore, scores differed significantly between Egypt and Saudi, Egypt and Sudan, and Egypt and Libya. These differences could be accounted for by other causative factors to fears in different countries (Al‐ghzawi et al., 2014). Some of these causative factors could be the changes in authority and the poverty in Egypt and Lebanon, which are demonstrated in exaggeration among these countries compared to the other study countries (Abdelhalim, 2018; Dunne, 2020). The other cause of this variance could be explained by the higher percentages of health care professionals among the Egyptian sample (44.5%), which are considered front liners in this battle, compared to the samples of the other countries. In terms of other sample characteristics, it was previously reported that female gender and younger age were associated with higher knowledge about the coronavirus (Hezima et al., 2020). This aligned with the current study's findings where the majority had good to excellent knowledge about the COVID‐19 spread and prevention 71% and 76.5%, respectively.

Additionally, the majority of the study sample were females (60.5%), educated (71.8% have bachelor or higher degree), and aged below 30 years (58.7%). This finding was complemented by another study conducted in Egypt, which showed that females and younger ages exhibited good knowledge about coronavirus diseases signs, spread, and prevention (Abdelhafiz et al., 2020). However, it was reported that females with low income and those having children below the age of 18 scored higher on the Fear Scale (Caycho‐Rodríguez et al., 2020), which complements the findings of our study.

Interestingly, small percentages of participants were diagnosed with respiratory diseases, prediabetes, diabetes, and autoimmune disease (1.9%, 1.8%, 3.1%, and 2%, respectively), while 6% were diagnosed with anxiety (Egypt the highest 8% and Sudan the lowest 3.1%) and 3.4% had insomnia (Egypt the highest 8.5% and Sudan the lowest 1.9%). Only 4.3% (*n* = 121) of the respondents suffered from depression, and 34 were from Egypt. Asmundson et al. (2020) reported a positive correlation between mental health, anxiety, and a fear score of COVID‐19.

The limitations of the current study lie in the interpretation of the fear scores of the study participants. Some occurrences of the countries, such as the Beirut blast, the floods in Sudan, and the civil war in Libya, coincide with the study timing and may have contributed to the results. Future studies should address the causes of the fear and nail down the triggers specific to every country at the study time. Additionally, future studies should dwell on the clinical practices and health care and mental health care availabilities in each country to provide recommendations specific to each setting. Another limitation is the availability of other Fear Scales that have been previously validated in the Arabic world. However, none of these scales specifically addressed fear in the COVID‐19 pandemic.

Additionally, this short scale of eight items makes it easy for participants to complete compared to the long Fear Scales used in the Arab region, including up to 120 items. Another limitation lies in the sample of the study, with the majority being younger ages. This should be addressed in future studies with different age groups. Additionally, future development of the Fear Scale should aim to identify a cut‐off score indicating fear or no fear.

## 5 CONCLUSION

Assessing the impact of the COVID‐19 pandemic on fear and health among Arab countries is critical to prevent the psychological outcomes of any pandemic diseases by following effective measures. The current study's findings indicate high levels of fear among the study participants, especially in Egypt and Lebanon. The Fear Scale has shown good psychometric properties when evaluated in five Arabic‐speaking countries and can be used in future studies. The future direction uses the scale among different age groups to verify the effect of fear on healthy middle‐aged and older adults.

### PEER REVIEW

The peer review history for this article is available at https://publons.com/publon/10.1002/brb3.2375

## Supporting information



Supporting Information

## Data Availability

Data sharing agreement: All data will be made available to facilitate research reproducibility while protecting patient privacy and confidentiality.
