# Supplementary material for: A multi‐centric study on validation of the Fear Scale for COVID‐19 in five Arabic speaking countries
Source: Brain Behav. 2021 Oct 17;11(11):e2375. doi: 10.1002/brb3.2375 (PMC8613427; doi:10.1002/brb3.2375)
Supplement: Supplementary file 1 — Supporting Information [file BRB3-11-e2375-s001.docx]

Appendix A

Arabic Fear Scale

مقياس الخوف

|  | أرفض بشدة | أرفض | محايد | أوافق | أوافق بشدة |
| --- | --- | --- | --- | --- | --- |
| ۱. إن فكرة كوفيد-19 تخيفني. |  |  |  |  |  |
| ۲عندما أفكر بكوفيد-19، أصبح عصبيا. |  |  |  |  |  |
| ۳. عندما أفكر بكوفيد-19، أصبح مضطربا. |  |  |  |  |  |
| ٤. عندما أفكر بكوفيد-19، أصبح كئيبا. |  |  |  |  |  |
| ٥. عندما أفكر بكوفيد-19، أصبح غاضبا. |  |  |  |  |  |
| ٦. عندما أفكر بكوفيد-19، قلبي ينبض أسرع. |  |  |  |  |  |
| ٧. عندما أفكر بكوفيد-19، أشعر بعدم الراحة. |  |  |  |  |  |
| ۸. عندما أفكر بكوفيد-19، أشعر بالتوتر. |  |  |  |  |  |
